# EFFECTS OF SOCIAL ENGAGEMENT ON WELL-BEING AND COGNITIVE FUNCTIONING IN OLDER HISPANICS

**DOI:** 10.1093/geroni/igad104.2187

**Published:** 2023-12-21

**Authors:** Hsiao-Wen Liao, Hyewon Kang

**Affiliations:** Georgia Institute of Technology, Atlanta, Georgia, United States; Georgia Institute of Technology, Atlanta, Georgia, United States

## Abstract

Research on engaged lifestyles demonstrates the importance of social engagement for aging well. The present study examined this thesis in the context of minority aging. With the theoretical lens that Hispanic cultures value social relationships, we tested whether social engagement was particularly important for maintaining well-being and cognitive functioning in Foreign-born (FB-H) and U.S.-born Hispanics (USB-H) relative to non-Hispanic Whites (NHW). Participants (N = 2677) was drawn from older adults in the Health and Retirement Study who completed the 2008, 2012, 2016, and 2020 module at least once. Two types of social engagement were assessed. Social interactions were based on the frequency of interactions with close partners (i.e., children, family members, and friends). Social activity participation was measured by the frequency of volunteering, doing charity, joining clubs, and attending meetings of non-religious organizations. Results of random intercept linear models controlling for covariates showed that, consistent with the literature, social interactions predicted better life satisfaction and that both aspects of social engagement predicted better immediate and delayed recall. Unexpected patterns emerged when interaction effects of race/nativity and social engagement were tested. While the general pattern held for NHWs, we found that greater social interactions predicted lower life satisfaction in USB-H. For FB-H, greater participation in social activities correlated with lower performance on immediate recall. Echoing a recent study that reveals mixed evidence for the well-documented paradox that Hispanic immigrants hold health advantages (Tarraf et al., 2020), we call for more research on lifestyle factors and aging trajectories in racially diverse populations.

